# Dissemination strategies for the national scale-up of a school-based physical activity initiative: insights from education and health sector partner organizations

**DOI:** 10.1186/s12889-026-28517-4

**Published:** 2026-07-14

**Authors:** Anna Fitriani, Ana María Contardo Ayala, Harriet Koorts, Jo Salmon

**Affiliations:** Institute for Physical Activity and Nutrition, School of Exercise and Nutrition Sciences, Deakin University, Burwood, VIC Australia

**Keywords:** Dissemination, School-based initiative, Physical activity, Sedentary behavior, Partnership

## Abstract

**Background:**

Whole-of-school physical activity initiatives have strong potential for population-level impact, yet few have been disseminated or scaled nationally. Given the relative lack of *dissemination* research compared with *implementation* research in this field, and the limited exploration of third-party perspectives, this study aimed to examine how cross-sector partner organizations disseminated *TransformUs Secondary* to schools across Australia.

**Methods:**

This qualitative study was incorporated into the *TransformUs Secondary* type 2 hybrid implementation–effectiveness trial. Sixteen partner organizations were invited to participate in online semi-structured interviews conducted 16-months post-launch. Interview transcripts were analyzed using reflexive thematic analysis, with findings organized into three major themes: dissemination strategies (types, frequency, pathways), barriers and enablers influencing partners’ dissemination efforts, and mechanisms underlying perceived effective dissemination strategies.

**Results:**

Out of 16 invited partner organizations representing diverse sectors, 10 participated in interviews. Partners employed multiple dissemination strategies, including different types (i.e. material development and distribution, educational meetings, and network mobilization), frequency (i.e. ranging from one-off email blasts to ongoing dissemination), and pathways (i.e. education-sector direct school engagement versus health-sector intermediary-led dissemination, each characterized by distinct channels and message framing). Partners’ dissemination efforts were shaped by teacher-level factors (perceived complexity, relevance, workload pressures), partner organizational factors (bureaucratic processes, role boundaries, limited capacity), and system-level factors (funding and competing priorities). Partners perceived that effective dissemination required not only access to resources, but also strategies that built implementer capacity, strengthened positive attitudes, and fostered school-level engagement.

**Conclusions:**

These findings are novel in highlighting how national dissemination of a school-based physical activity initiative operates across diverse partners, showing that dissemination pathways, channels, and messages are shaped by the actors involved, and extend beyond simply sharing materials. Future research should design and test dissemination strategies, as well as explore how they interact with contextual barriers to optimize dissemination outcomes. Policy should support partner organizations to navigate organizational and system challenges, while researchers strengthen partner dissemination efforts.

**Trial registration:**

This study was prospectively registered with the Australian New Zealand Clinical Trials Registry (ANZCTR) under the identifier ACTRN12622000600741 on 21 April 2022.

**Supplementary Information:**

The online version contains supplementary material available at https://doi.org/10.1186/s12889-026-28517-4.

## Contributions to the literature

- Unlike previous school-based physical activity studies that primarily focused on *implementation*, this study shifts attention to *dissemination*, demonstrating how an initiative was disseminated at a national scale through third-party organizations.
- While existing literature presents a relatively narrow range of dissemination strategy types, partners in this study used a broader and more practical set of strategies.
- In contrast to literature depicting simple, linear frameworks, dissemination in practice was complex, involving chained pathways and actors with varying capacities, roles, and mechanisms of influence.
- Although research–practice partnerships are recommended to support scale-up, barriers often arose within partner organizations, a finding that few studies in this field have highlighted.
- Overall, this study provides insights into how dissemination strategies are employed in real-world, highlighting the need for guidance and policies to support large-scale dissemination involving partner organizations.

## Introduction

Scaling up (i.e. the horizontal expansion of an initiative to reach a wider population and benefit more people, or vertical integration into policy systems [1]) evidence-based whole-of-school initiatives for promoting physical activity and reducing sedentary behavior among adolescents has been recommended to address these widespread behaviors among young people [2, 3]. Dissemination (i.e. the active process of spreading initiative information and resources to target audiences through specified channels [4]), which is an essential early stage of scale-up, aims to increase awareness of and intention to adopt an initiative [5]. During national scale-up, dissemination becomes more challenging due to the need to engage diverse education systems, stakeholders, and contexts [6]. Dissemination strategies, involving the development and distribution of messages and materials to target audiences, can help support these efforts [7].

While cross sector government and non-government partner organizations commonly support dissemination by bridging researchers and schools [8, 9] through their local knowledge, resources, and networks [10–12], their role in dissemination remains conceptually underdeveloped. Leeman et al.’s framework recognizes that dissemination strategies may be undertaken by actors across multiple systems, including partner organizations as the support system [7]. Although researcher-led dissemination strategies have been documented in the *TransformUs Secondary* scale-up [13], partner-led dissemination (e.g., types and frequency of strategies used) remain poorly understood. Brownson et al.‘s model for dissemination research conceptualizes dissemination as a pathway linking a source, channels, and target audiences [14]. While researcher-led dissemination pathways have recently been described [13], how these pathways operate when partner organizations act as the dissemination source remains unclear. Although barriers of implementation (i.e. the process of putting an evidence-based initiative into practice in particular settings [15]) have been conceptualized (e.g. Ecological Framework [16]), comparatively little attention has been given to factors influencing dissemination processes, particularly those involving intermediary organizations. Similarly, while growing research has examined the mechanisms through which implementation strategies influence outcomes [17], understanding of the mechanisms underpinning dissemination strategies remains limited.

As intermediary actors positioned between researchers and schools, understanding their perspectives on how dissemination strategies reach schools, including barriers and mechanisms influencing adoption, is important for supporting their dissemination efforts and improving the design of future dissemination strategies. While previous school-based physical activity studies have reported experiences of partnership-based dissemination, including challenges in maintaining partner buy-in over time [18], limited partner capacity and insufficient school connections [19], and the resource-intensive nature of coordinating dissemination activities with partners [20], limited insight remains into how partner-led dissemination operates in practice and how it influences adoption in school settings. This information is necessary to understand which strategies can be learned from and where gaps exist, to refine support and coordination and ensure evidence-based initiatives are effectively scaled and integrated across secondary school contexts.

This study addresses this gap by investigating dissemination strategies used by partner organizations to facilitate teacher awareness, engagement, and adoption of *TransformUs Secondary*, a national school-based physical activity and sedentary behavior initiative designed to support teachers in integrating movement into classroom practice and school environments across Australia. Specifically, the study aimed to: (1) explore the dissemination strategies used by partners; (2) explore the perceived factors influencing dissemination and scale-up efforts; and (3) examine the mechanisms through which dissemination strategies potentially contributed to teacher awareness, engagement, and adoption of the initiative.

## Methods

### Initiative and setting

*TransformUs* is an evidence-based, whole-of-school initiative to promote students’ physical activity and reduce sedentary behavior that aligns with the Australian curriculum [21]. The initiative supports teachers in embedding movement into regular lessons (e.g., Mathematics and English) and modifying the school environment to foster more movement-friendly spaces [21].

The *TransformUs* primary school initiative is currently being implemented at scale in Australia [21], and *TransformUs Secondary* has been adapted and tested for adolescents in secondary school settings [22]. *TransformUs Secondary* consists of six distinct components: (1) active academic lessons, (2) active breaks, (3) active homework, (4) active school environments, (5) health lessons, and (6) peer support [22]. To support scale-up, initiative professional learning and resources are available on the *TransformUs* website, ensuring accessible online access for users nationwide.

*TransformUs Secondary* was scaled up nationally in October 2023, as part of a Type 2 hybrid implementation–effectiveness trial, which assesses both initiative effectiveness and strategies to support implementation [22]. For the implementation trial, all secondary schools across Australia (*n* = 1,453) and their staff could register online to access the initiative [22]. Registered schools and staff have the option to either use the initiative without participating in the research or take part in the research component of the implementation trial by completing baseline surveys, implementing the initiative, and providing follow-up feedback after six months of initiative use and implementation [22].

In the initial stage of scale-up, *TransformUs Secondary* was disseminated to increase school staff awareness, encourage registration on the initiative website, and promote ongoing engagement (e.g., accessing free professional development and initiative resources) with the goal of supporting routine adoption in daily school practice. The *TransformUs Secondary* research team engaged 16 partner organizations (e.g., state departments of education and health, professional teaching organizations) to facilitate dissemination and promote the initiative to schools and teachers nationwide [13]. To facilitate this, the *TransformUs* team developed a dissemination toolkit with adaptable messaging (e.g., newsletters, social media) for partner organizations, alongside social and mass media promotions to reach school staff directly (example of pages can be found in Supplementary file 1). Beyond dissemination, partner organizations were also requested to support scale-up of the initiative by leveraging their organizational capacity (e.g., funding, policy influence, and sectoral networks) to promote integration and sustainment of *TransformUs Secondary* into the school system.

### Study design and participants

The current qualitative study was conducted as part of the type 2 hybrid effectiveness-implementation trial [22]. Sixteen months after the launch of *TransformUs Secondary*, representatives from the 16 partner organizations were purposively invited to participate in online semi-structured interviews. Eligibility included representatives from each of the 16 partner organizations who had prior contact with the *TransformUs* team, and who were invited to support dissemination of *TransformUs*, engaged in related dissemination discussions, or attended follow-up meetings after the initiative’s launch. Participants were included regardless of their level of involvement. Participants represented a range of intermediary organizations involved in dissemination of *TransformUs Secondary*, including government and non-government organizations from the education and health sectors, with roles spanning education, health promotion, and wellbeing, and varying levels of experience in dissemination and scale-up activities. This allowed the study to capture both the experiences of those actively engaged in dissemination and those with limited or no involvement. Participants were recruited via email and provided signed informed consent prior to participation. They were informed of the study aims and objectives prior to the interview, and that the interviewer (AF) was a PhD candidate within the *TransformUs* project. This study was reported in accordance with the Consolidated Criteria for Reporting Qualitative Research checklist [23] (Supplementary file 2).

### Semi-structured interview

One-on-one interviews were conducted via video conferencing between March and April 2025 by AF, a female PhD candidate with prior experience in qualitative research and interviewing and had no prior relationship with participants. The interview guide was developed by AF with input from the research team, JS (Distinguished Professor), HK (Associate Professor), and AMCA (Postdoctoral Research Fellow), all of whom were experienced researchers with formal training and expertise in qualitative research and implementation science. AF approached the study from an implementation science and public health perspective, with a focus on understanding dissemination of school-based physical activity initiatives for adolescents health and wellbeing.

While this study focused on dissemination, partners’ broader experiences with scale-up were also explored, given their role in facilitating broader initiative integration. The interview guide was developed based on the Reach, Effectiveness, Adoption, Implementation, and Maintenance (RE-AIM) framework [24], beginning with questions on strategies and factors influencing Reach (dissemination), and progressing to broader scale-up factors affecting initiative integration into routine school practice (Adoption, Implementation, and Maintenance) (see interview schedule in Supplementary file 3). The theoretical frameworks used throughout the study, including their purpose and rationale for use, are summarized in Table 1.

**Table 1:** Theoretical frameworks applied in this study

Framework	Purpose	Rationale for use
The RE-AIM framework	To guide the interview questions	The framework was used to ensure comprehensive exploration of key dimensions relevant to scale-up, including dissemination.
School Implementation Strategies, Translating Expert Recommendations for Implementing Change Resources (SISTER) [25] and Leeman et al.’s dissemination framework[7]	To classify types of strategies used to enhance consistency and comparability	The SISTER framework provides some strategy types related to dissemination (e.g. *use mass media*, *distribute message and material*), while Leeman et al.’s framework offered overarching dissemination strategy classifications (i.e. message and material development and distribution). These frameworks were combined to enable comprehensive coding of partner-led dissemination strategies.
Brownson et al.’s framework for dissemination of research [4]	To clarify dissemination pathways	The framework provides a structured dissemination pathway linking source, message, channel, and target audience, serving as a foundation for examining and extending dissemination pathways when various actors acted as the dissemination source.
Ecological Framework [16]	To capture dissemination and scale-up factors	The framework provides a multi-level structure (e.g. school, community, and support system) levels for organizing and interpreting factors influencing dissemination and scale-up across.
Behavior Change Wheel [26]	To identify mechanisms through which perceived effective strategies influence desired outcomes	The framework links dissemination strategies mapped as intervention function to targeted behavioral determinants (e.g., access to resources, perceptions, and skills) and intended outcomes (e.g. awareness, motivation, and intention to adopt).

Each interview began with questions about the participant’s role within their organization, and their experiences with the dissemination and scale-up of *TransformUs Secondary (e.g.* strategies used, perceived barriers and enablers, and which strategies they considered most effective and why). For participants who had not been involved in the dissemination of *TransformUs Secondary*, or had done so to a minimal extent, the interview focused on exploring the reasons for their limited involvement, including barriers that hindered their participation. These participants were also asked to reflect on effective dissemination strategies for school-based physical activity initiatives more broadly, drawing on their experience with other initiatives. Each interview lasted approximately 30 min and was audio-recorded with consent. Only the participant and the interviewer were present during the interviews. Field notes were made during and after the interviews to capture contextual details, non-verbal cues, and reflections. No repeat interviews were undertaken, and transcripts were not returned to participants for comment or correction. During familiarization and coding of each interview, recurring patterns in dissemination strategies, barriers, and enablers became evident within and across education and health-sector partners. As additional interviews were conducted, the data largely confirmed existing patterns rather than contributing substantively new concepts, suggesting that sufficient depth of information had been obtained to address the research aims.

### Data coding and analysis

Interviews were transcribed verbatim and analyzed using NVivo 15, guided by the six phases of reflexive thematic analysis [27]. Initial familiarization involved repeated reading of transcripts and noting preliminary observations about partner organizations’ experiences with dissemination and scale-up. Reflexive notes were also taken throughout this process, capturing insights and impressions, such as where some partners appeared less actively engaged in dissemination and scale-up activities. The interview data were initially coded by AF in an iterative and flexible process, involving close engagement with the data. Coding was both deductive and inductive: deductive coding was informed by relevant frameworks (Table 1), used as sensitizing concepts rather than fixed coding categories, while inductive coding captured emergent patterns not fully accounted for by these frameworks. Codes were repeatedly revised, renamed, merged, and refined throughout analysis as interpretations developed.

To distinguish dissemination from broader scale-up experiences, dissemination was conceptualized using Leeman et al.’s framework as a guiding interpretive lens, focusing on activities involving the development and distribution of messages and materials, while activities primarily supporting initiative implementation or integration within schools were classified as scale-up-related [7]. To describe the mechanisms by which perceived effectiveness of dissemination and scale-up strategies may influence school staff engagement and adoption, interview data were interpreted through the Behavior Change Wheel as an analytic framework [26]. This framework was used as an interpretive lens to examine how participants described potential mechanisms linking dissemination strategies, behavioral responses, and anticipated outcomes.

Coding and theme development were primarily undertaken by AF, with regular discussions with JS, HK, and AMCA. These discussions were used to critically reflect on emerging interpretations, refine conceptual boundaries, and deepen analytic insight. Where differences in interpretation arose, they were explored through dialogue and revisiting the data, which contributed to refinement and further development of analytic interpretations. Finally, potential themes were developed by examining patterns of shared meaning across codes in relation to the research questions, iteratively reviewed, refined, and defined to ensure coherence and distinctiveness. The results are organized into three major themes based on the study aims: dissemination strategies; barriers and enablers for dissemination and scale-up; and mechanisms underlying perceived effective dissemination and scale-up strategies (Summary of themes and sub-themes can be seen in the supplementary file 4).

## Results

### Sample characteristics

Interviews were conducted with representatives from 10 of the 16 organizations involved in supporting the dissemination of *TransformUs Secondary* across Australia. The remaining six organizations did not participate due to reasons such as limited involvement in the initiative (*n* = 2) or non-response to the invitation to be interviewed (*n* = 4). A summary of the participants’ characteristics is provided in Table 2. Participants represented a range of organizations, including State Education Departments, State Health Departments, professional teaching associations, and non-government organizations from the education and health sectors. The ten participating organizations were based across five of the six Australian states and two territories, including Victoria, Northern Territory, Queensland, New South Wales, and South Australia. A professional teaching organization was based in Victoria but is accessed by teachers nationally. Of the 10 partners who took part, some partners (*n* = 2) had not yet been involved in dissemination or had only limited experience (*n* = 1), and none had taken steps to support the initiative’s scale-up, such as allocating organizational priority or funding to facilitate dissemination activities.

**Table 2:** Participant characteristics

No of participants	Organization type	Organization Code^*^	Role	Role duration
4	Government education authority	A	Education and wellbeing professionals	Less than 15 years
2	Government health authority	B	Health promotion professionals	Up to 20 years
2	Professional teaching associations	C	Education professionals	More than 20 years
2	Non-government organizations from the education and health sectors	D	Wellbeing and health professionals	Less than 15 years

*Organization codes (A–D) are used to facilitate referencing in quotes

### Theme 1: dissemination strategies for *TransformUs secondary*

Across partners involved in the dissemination of *TransformUs Secondary* (*n* = 7), a variety of strategies were employed, with some partners (*n* = 5) implementing more than one strategy. These strategies included developing promotional materials, distributing information and resources through routine communication channels (e.g., emails, newsletters, flyers, teacher e-learning platforms), conducting education meetings (teacher professional development sessions and stakeholder meetings), and leveraging professional networks and liaisons. Some partners created their own materials: *‘We’re developing our own resources*,* one-page flyers with simple activities teachers can use at lunchtime’* (Participant 1, organization A).

Distribution strategies varied, ranging from simple approaches such as email blasts to more creative methods, including integrating the initiative into a widely used secondary school physical education textbook: *‘I’ve actually put a TransformUs article in a year 9 and 10 health book*,*’* (Participant 2, Organization C). While some partners reported primarily distributing information and resources, others conducted interactive engagement educational meetings. Education-sector partners engaged teachers by demonstrating *TransformUs Secondary* resources during a professional development session, while health-sector partners engaged local organizations through stakeholder meetings. Most leveraged existing professional and organizational networks, such as public health or teacher associations, to extend initiative visibility. These networks were perceived as trusted and effective for amplifying dissemination, with local intermediaries (e.g., regional sports coordinators, local government staff) often relaying information to schools when partners could not directly engage with teachers. Further examples of dissemination strategies, along with additional illustrative quotes, are provided in Supplementary file 5.

**Fig. 1:**
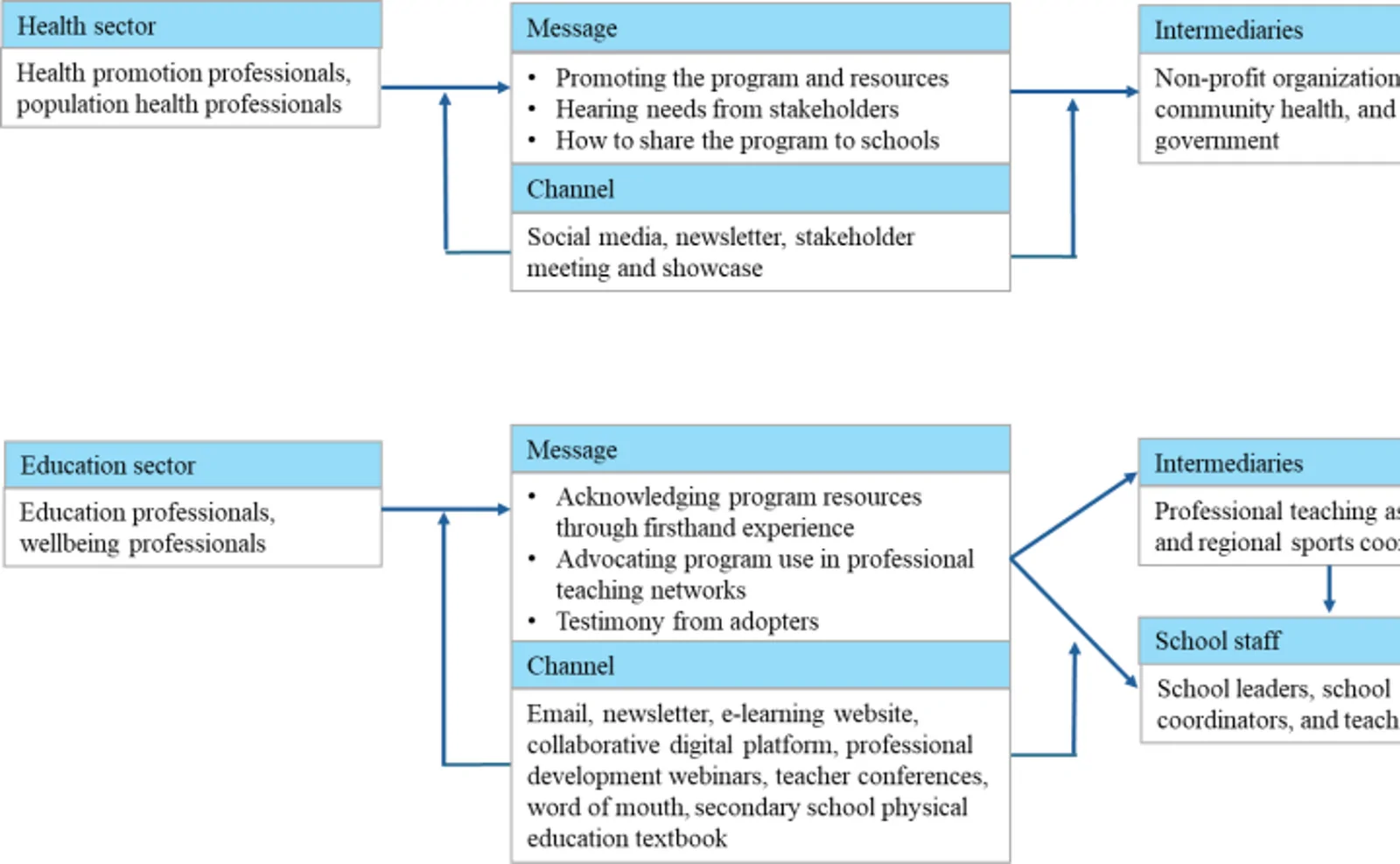
Dissemination pathway of *TransformUs Secondary* from partner organization to school staff. Dissemination activities described by participants were mapped onto an adapted version of Brownson et al.’s framework for dissemination of research [4], illustrating how strategies flowed from partner organizations to school staff

Dissemination pathways reflected sectoral differences (Fig. 1). While education-sector partners engaged school staff directly, health-sector partners typically relied on intermediary stakeholders, such as regional health officers, to cascade information to schools. Although some education-sector partners also engaged intermediaries, these were closely connected to schools, such as professional teaching associations and regional sports coordinators. These differences were not merely operational choices but reflected structurally determined roles, mandates, and access pathways that shaped how dissemination could be enacted across sectors. Education-sector partners, whose core role involves direct engagement with schools through teacher professional development and curriculum support, were structurally mandated to work within school systems. This positioning enabled them to directly access teachers and embed *TransformUs Secondary* within existing professional learning structures, making dissemination a routine extension of their work. In contrast, health-sector partners primarily operated within public health promotion and prevention mandates focused on population-level outcomes. This role did not include direct responsibility for curriculum delivery or routine school engagement, resulting in more limited legitimacy and access to classrooms. Consequently, dissemination was more indirect and cautious, relying on intermediary networks and stakeholder pathways to reach schools.

These structural differences between the education and health sectors were also reflected in the framing of dissemination messages and the choice of channels (Fig. 1). Messages delivered by the education-sector partners focused on firsthand experiences, highlighting the practical use of resources and adopter testimonies. Meanwhile, health-sector partners emphasized introducing the initiative’s relevance to local stakeholder needs and how it might be conveyed to schools. Dissemination channels used by the education-sector partners were more diverse and embedded within teachers’ usual platforms, such as newsletters, e-learning websites, collaborative digital platforms, and professional development webinars. Health-sector partners, by comparison, used fewer channels, primarily directed at intermediaries.

The frequency of dissemination varied across partners based on channels used. Emails were used sporadically, primarily for initial initiative promotion, while newsletters were distributed more regularly (bi-monthly to quarterly), with *TransformUs Secondary* content included when new resources were available. Some partners went beyond emails and newsletters by delivering professional development sessions that consistently incorporated and promoted *TransformUs Secondary* resources (e.g., displaying materials during events). These professional development sessions were delivered multiple times per year based on teachers’ needs, providing opportunities for deeper engagement: *‘We run multiple trainings throughout the year … up to a dozen sessions’* (Participant 3, organization A). Some partners (*n* = 3) integrated *TransformUs Secondary* into websites and textbooks, ensuring sustained visibility beyond one-off dissemination activities.

### Theme 2: dissemination barriers and enablers within the broader scale-up context

Partners described a range of factors influencing successful dissemination, including school-level factors (e.g., staff attitudes and time constraints) and partner-related factors (e.g., organizational capacity and research team support). In addition, broader scale-up factors, such as funding limitations and competing priorities, were identified as contextual conditions that influenced partners’ dissemination efforts. Teachers’ perspectives about the initiative emerged as both a critical enabler and barrier to dissemination.

While *TransformUs Secondary* was generally regarded as beneficial and accessible, one partner described how teachers perceived the initiative’s resources as overly complex in their packaging and presentation, which discouraged initial engagement and promotion, particularly in the context of existing workload pressures: *‘One of the barriers is the complexity that teachers see in the resources. You know*,* a number of layers there*,* it looks like it just looks and feels like it’s quite big. And I think that in itself might be a deterrent for some*,* in that it might look overwhelming to even start.’* (Participant 1, organization A). This perceived complexity may have created an initial engagement barrier, particularly given that *TransformUs Secondary* was promoted as a package of training and resources accessed through multiple layers of website content. Workload pressures compounded these perspectives, as heavy teaching and administrative demands left teachers with little time to meaningfully engage with initiative resources or attend professional learning where the initiative was promoted. As one participant reflected, *‘You can promote and advertise and talk about it till the cows come home*,* but it really depends on people in schools actually taking it up. [A] barrier is getting people to really dig in and look at it. We always have barriers to getting people to our professional learning as well’* (Participant 3, organization A).

Resistance was also rooted in pedagogical beliefs. As one participant explained, ‘*It’s more of a problem in secondary schools because they think kids in secondary schools tend to think of it as a primary school initiative’* (Participant 1, organization A). This perception may have been reinforced by the fact that *TransformUs* was originally developed and implemented in primary school settings. Although *TransformUs Secondary* was adapted for adolescents and emphasized movement integration within regular secondary school teaching practices, teachers who were familiar with the primary school initiative may have continued to associate the initiative with younger children. In addition, resistance reflected broader beliefs about teaching and learning: *‘Teachers in learning areas other than health and physical education don’t value or view a movement-based pedagogy as a meaningful and impactful pedagogical way of learning’* (Participant 1, organization A). This resistance may stem from a perceived tension between curriculum delivery and movement integration. For teachers working in academically intensive subjects, allocating class time to movement-based activities may be viewed as detracting from instructional goals, particularly when the cognitive and educational benefits of such approaches are not well understood.

Partners’ dissemination efforts were shaped by organizational factors and the extent of research team support available. Dissemination was easier when the initiative matched organizational values and priorities focused on health, wellbeing, and student engagement. Access to established teacher networks and digital platforms also enabled partners to reach schools more effectively. Yet dissemination was also shaped by organizational structures that constrained the flow of information both within and across partner organizations: ‘*It’s very hard to get things approved to disseminate information. It has to meet a lot of kind of communication guidelines and standards. Even we find it difficult to communicate about our initiatives in the department*,* given how complex and big the department is’* (Participant 4, Organization A). Structural role boundaries created further obstacles, as partner organizations were often restricted to their own jurisdictional remit, limiting opportunities for cross-sector collaboration: *‘I can’t easily share it beyond this department space [to other sectors] because it has such tight restrictions and we can’t share our materials beyond our own cohort of employees’* (Participant 6, Organization A). These findings suggest that dissemination was influenced not only by partners’ willingness to promote the initiative but also by organizational communication systems that constrained the speed and flexibility of information sharing. While approval procedures ensured alignment with departmental priorities and communication standards, they could inadvertently slow dissemination efforts and reduce momentum by limiting responsiveness to emerging opportunities. However, these challenges appeared to reflect broader organizational communication processes rather than issues specific to *TransformUs Secondary*.

Dissemination capacity also varied across organizations. Some organizations had dedicated staff or teams, while others operated on volunteer-based efforts with little expertise in physical activity promotion: *‘We were just really exploring TransformUs as an opportunity like as a good initiative to be introduced to schools because we don’t have necessarily any expertise around that’* (Participant 8, organization D). Therefore, the research team support is important for them. For example, the dissemination toolkit developed by the research team was considered a crucial support, providing partners with consistent, evidence-based messaging and materials that carried institutional credibility. However, some highlighted the need for more tailored resources, visual content, and testimonials to strengthen the toolkit’s appeal. Partners emphasized the need for ongoing engagement and support from the *TransformUs* team to maintain momentum and keep dissemination on their agenda: *‘I wouldn’t have advertised TransformUs for a while. More than happy if there’s any new*,* especially findings from research*’ (Participant 3, organization A).

At a broader system level, partners consistently emphasized the constraints of limited funding and competing policy priorities on efforts to scale-up *TransformUs Secondary*, providing important context for dissemination efforts. During interviews, when questions shifted from dissemination to scale-up, participants were asked about deliberate actions to embed the initiative into school systems (e.g., securing funding or influencing policy) and they indicated that such efforts had not yet occurred. In many cases, there were no sustainable financial mechanisms to support large-scale integration, with some agencies relying on one-off budget underspends to deliver related initiatives. These funding constraints provided important context for the dissemination of *TransformUs Secondary* and the capacity of partner organizations to support its promotion over time. As some participants explained: *‘Any resource like the funding that scaled up was a one-off funding because they actually had an underspend close to the end of a financial year*,* and we entered that partnership as a one-off situation. And as far as scaling goes*,* we’re trying to initiate a initiative of our own’* (Participant 6, organization A); *‘It sounds like we had these intentions to move forward with it (scale-up)*,* but I believe there may have been a funding limitation that kind of came into the play’* (Participant 5, organization B). Even where there was interest in scaling up, and evidence is clear, other agendas often took precedence, particularly healthy eating initiatives, while within education, system leaders placed strong emphasis on literacy and numeracy targets. As a result, physical activity initiatives often faced challenges gaining attention and priority within dissemination efforts, despite clear evidence of their benefits. As one partner observed: *‘They continually just repetitively require compliance and focus on literacy … the research and the evidence is absolutely there*,* and it just gets continually ignored’* (Participant 6, organization A).

### Theme 3: perceived mechanisms linking dissemination strategies and outcomes

**Fig. 2:**
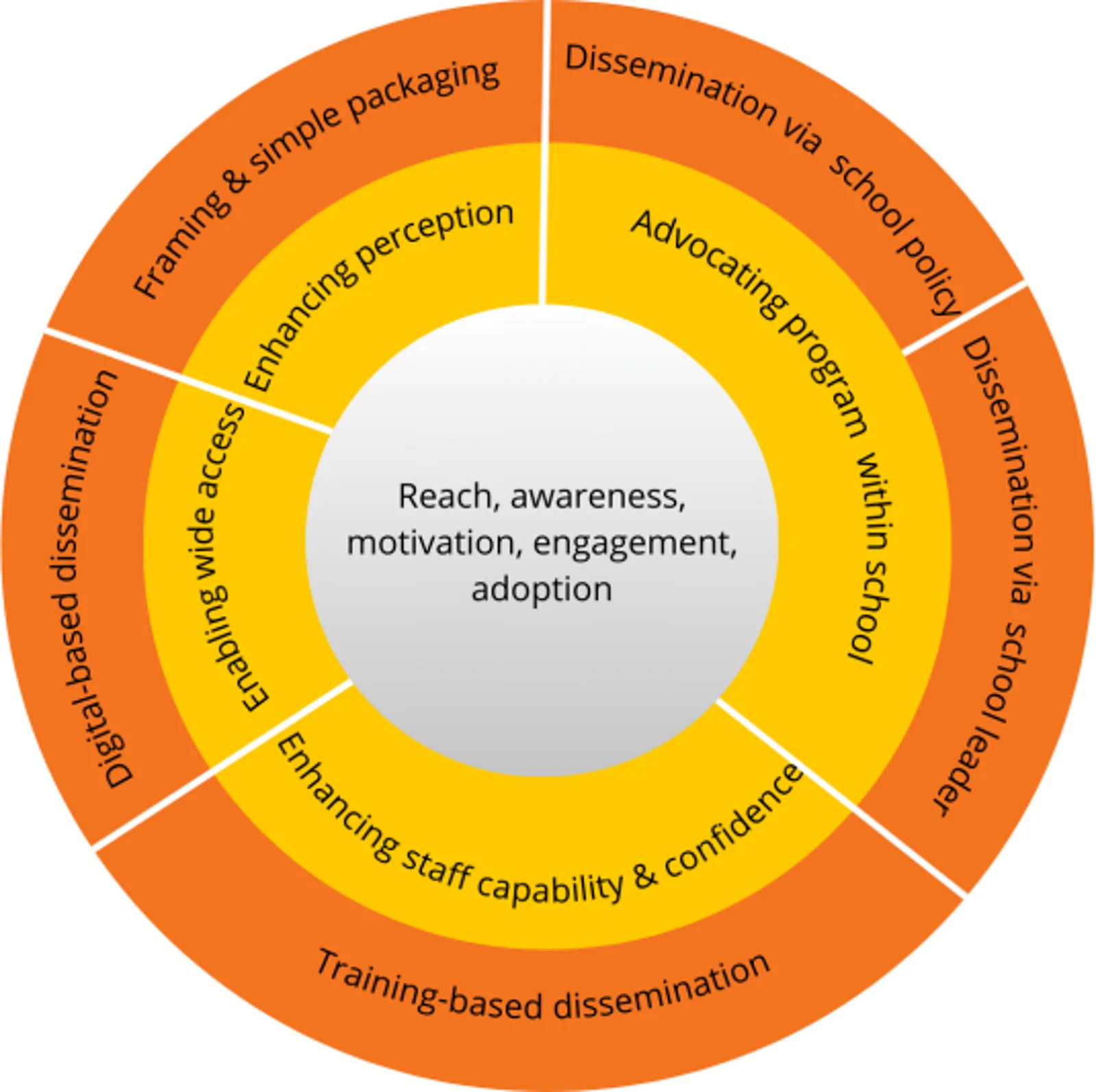
Mechanisms through which perceived effective dissemination strategies influence dissemination outcomes. This framework illustrates how perceived effective dissemination strategies (orange outer ring) and strategies functions (yellow middle ring) interact to potentially influence dissemination outcomes (center), as derived from the interview transcript

Partners described which dissemination strategies they considered most important, based on their experiences and expectations, and how these strategies were perceived to potentially influence dissemination outcomes (Fig. 2). These perceptions were mapped onto the Behavior Change Wheel [26] as an interpretive framework, where strategies (outer ring) were linked to functions (middle ring) and outcomes (inner ring) to help understand how participants believed dissemination efforts might support engagement and adoption. Overall, partners perceived that effective dissemination strategies must not only enable wide access to the initiative resources, but also build school staff capacity, enhancing their attitudes of the initiative, and increasing school level awareness and engagement.

Digital dissemination was perceived to support wider access to initiative resources and to contribute to broader awareness and reach. Digital platforms, such as state-level teacher e-learning systems, were described as enabling equitable access to resources for teachers who chose to engage. For example, partners noted placing materials under a ‘physical literacy’ tab allows any interested teacher to access them: ‘*I put it on our e-learn website*,* under physical literacy tab*,* so any teacher in the state can access that site’* (Participant 3, organization A). Such approaches were seen as potentially increasing visibility and accessibility, particularly for teachers already motivated to seek resources.

Training activities were perceived to contribute to building school staff capacity and, in some cases, may support motivation to adopt the initiative. For example, exposing teachers to *TransformUs Secondary* resources during professional development sessions was described as providing firsthand experience and exposure to practical activities: *‘Incorporating that into professional learning…people get that firsthand taste of an activity [active break)*,* is probably really the best way’* (Participant 3, organization A). Similarly, support for facilitators were viewed as potentially enabling schools to build internal capacity to implement active practices: *‘If there’s funding available*,* local government or community health could be targeted to build the capacity of facilitators within schools’* (Participant 10, organization B).

Strategic framing and simplifying resources into manageable components were perceived to potentially influence school staff attitudes and willingness to engage with the initiative. Partners emphasized framing *TransformUs Secondary* in ways that highlighted perceived relevance to both student wellbeing and academic outcomes. One participant noted the value of demonstrating links to academic achievement: ‘*If you could demonstrate a direct link between TransformUs and academic outcomes*,* that’s helpful. If you can do the same with student wellbeing*,* that’s a good thing’* (Participant 1, organization A). Simplifying materials and reducing perceived complexity were also seen as ways to make initial engagement more achievable, particularly when teachers were time-constrained: *The little snippets of professional learning are good too*,* because they don’t take up much time’* (Participant 3, organization A); *‘Keeping it relatively simple and achievable. I think just getting them involved initially*,* and then they can grow what they do after a period of time*,* they start very small*,* and it doesn’t feel overwhelming’* (Participant 1, organization A).

Finally, dissemination through school leadership and policy was perceived to potentially support school-level awareness and collective engagement with the initiative. Rather than targeting all staff simultaneously, participants suggested focusing on influential individuals such as school leaders or wellbeing coordinators who could support internal dissemination: ‘*I think about the target audience*,* which is really secondary school health and physical education teachers and maybe the leaders of the school*,* what would be in it for them to get involved?’* (Participant 7, organization D). Early adopters were also viewed as potential internal champions who could help coordinate and share information within schools: *‘You might have a dedicated coordinator*,* so the information comes in to that person and then they pass that on to the relevant people within the school*,* to make sure that the information is received’* (Participant 2, organization C). Supportive school policies were further described as potentially creating a conducive environment for engagement by aligning staff practices and expectations: *‘What we are trying to do is really get them like really work with schools*,* to work on things like the policy*,* like just getting policies and practices consistent and like that*,* everybody knows about it. And everybody in the school is on board because it requires the whole team committed to it’* (Participant 10, organization B).

## Discussion

This study investigated strategies used by partner organizations to disseminate a nationwide school-based physical activity and sedentary behavior initiative for adolescents. It also examined how these strategies were implemented, the barriers to dissemination and scale-up, and the mechanisms through which perceived effective strategies may influence school staff awareness, knowledge, skills, and attitudes. Findings indicated that: (1) dissemination involved diverse strategies, pathways, and frequency; (2) dissemination was influenced by school staff’ engagement, partners’ dissemination efforts, limited funding, and competing priorities; and (3) perceived successful dissemination required strategies that not only enhanced access to initiative resources, but also motivated teachers to engage, build their skills, and cultivate school-level engagement.

Partner organizations employed diverse dissemination strategies to reach schools and teachers, ranging from passive approaches such as emails and newsletters to more interactive methods like teacher professional development and stakeholder meetings. This reflects the real-world nature of dissemination, where some strategies are actively facilitated while others are diffused passively [28]. Some partners in this study employed interactive dissemination strategies, such as teacher professional development by education sector partners and stakeholder meetings by health sector partners, which may promote deeper engagement by allowing school staff and stakeholders to discuss barriers and initiative offerings [29]. Some education sector partners also enhanced school staff knowledge and skill acquisition by integrating examples of *TransformUs* resources into professional development, which may increase staff confidence to implement the initiative [11]. Partners’ active dissemination to engage school staff and build their skill are critical during scale-up, serving as an extension of the research team’s reach, which cannot feasibly cover all schools across multiple states. It has been highlighted that effective dissemination involves not only providing access and reach but also actively engaging target audiences to develop their understanding, attitudes, skills, and motivation to use the innovation/initiative [7, 30, 31]. Because adoption is inherently a cognitive and attitudinal process [32], passive distribution alone is insufficient [31]; a challenge during scale-up. This underscores the need to support partners in delivering interactive, engagement-oriented dissemination strategies.

Variations were also evident in dissemination pathways, channels, and frequency. Unlike the straightforward approach depicted in the proposed framework for dissemination research [4, 11], our partners’ dissemination in practice was complex, often requiring intermediaries to reach school staff and tailored channels to engage diverse audiences. This highlights the importance of establishing clear dissemination pathways [33] and cultivating strong networks to coordinate multiple actors (policymakers, stakeholders, and school community) [11]. Although there is no consensus on which channels are most effective or the optimal number and type of channels, evidence suggests that using multiple channels is associated with improved dissemination outcomes [34], highlighting the importance of adaptable toolkits to accommodate variations in channel use across partners. The frequency and sustainability of dissemination efforts undertaken by partners are also important, given that dissemination is an ongoing process that requires time and continuity [34]. Some partners in this study moved beyond one-off email communications to generate initial reach and awareness and instead engaged in ongoing dissemination through newsletters and websites. Although not assessed in the current study, this may help sustain initiative visibility and engagement over time. Thus, ongoing support from the research team (e.g. providing regular initiative updates for partner communications) may facilitate frequent and sustained dissemination efforts.

Although partners perceived that teachers recognized the initiative’s benefits and accessibility, teacher engagement with disseminated materials was influenced by attitudinal and contextual barriers. Attitudinal barriers are central targets of dissemination strategies, acknowledging that target audiences possess pre-existing knowledge and perceptions that fundamentally shape engagement with and responses to disseminated information [7, 11]. Attitudinal factors identified in this study (e.g. staff perceptions that physical activity initiatives are not suitable and not mandatory for adolescents, and perceptions of initiative resources as complex) are common in school-based physical activity initiatives, where perceived compatibility, need, and complexity have been shown to influence staff intention to adopt [35, 36]. Contextual barriers, including limited time among school staff to engage with and learn from disseminated resources, have also been identified as key challenges to dissemination across other audience groups (e.g. policymakers) [30]. Regardless how much dissemination effort was employed, if school staff lack the time to engage with and learn from these resources, the potential impact (e.g. knowledge and skill acquisition) is likely to be limited [37]. Therefore, dissemination strategies should aim to improve school staff attitudes while also reduce engagement barriers to initiative materials [5]. Framing the initiative around academic and wellbeing relevance, communicating implementation simplicity, and embedding resources within routine professional learning structures may represent practical mechanisms to enhance uptake.

While clear governance structures and adequate resourcing can facilitate dissemination [38], our findings suggest that overly rigid systems may create bottlenecks where bureaucratic processes and unclear role boundaries frequently constrained dissemination. Several partners also reported limited dissemination activity due to reliance on volunteers rather than dedicated staff, coupled with limited dissemination expertise and guidance, mirroring dissemination challenges in literature [39]. At the system level, insufficient funding and competing policy priorities constrained partners’ capacity to deliver and sustain dissemination activities during scale-up. This aligns with evidence that political and social climates strongly influence the initiation of scale-up in Australia [40]. These findings reflect the meso-level context, where organizational and intermediary system capacity (e.g. governance, role clarity, and administrative processes) shapes the feasibility of dissemination activities [41]. These challenges are often compounded when responsibility for dissemination shifts to partner organizations during scale-up [6]. Dissemination planning should therefore involve early collaborative planning with all relevant partners [6], alongside assessment of partner readiness and capacity, and differentiation of dissemination expectations according to organizational contexts [28]. This underscores the importance of advocacy and resource mobilization to support ongoing dissemination efforts during scale-up [40].

Partners described several mechanisms critical for targeting dissemination outcomes, including reach, staff attitudes and skills, and school-wide engagement. Reach can be enhanced by embedding initiative resources within existing teacher digital platforms, such as e-learning systems, enabling broad, low-cost, and sustainable dissemination across schools. Although the platform requires teacher login access, it aligns with Open Education Resource principles, supporting collaboration and free access [42]. Staff attitudes can be strengthened by strategically framing and simplifying initiative materials, emphasizing student wellbeing and academic outcomes, and highlighting evidence of initiative effectiveness, and strategies to increase motivation to engage [31, 43]. Staff skills and confidence can be developed via training-based dissemination, including professional learning workshops and hands-on demonstrations [30, 44]. School-wide awareness and climate, though rarely highlighted in existing dissemination frameworks, were recognized by partners as essential organization-level outcomes. Dissemination through school leaders and policy integration allowed principals, wellbeing coordinators, and lead teachers to amplify social opportunities and institutionalize initiative promotion, strengthening engagement across the school community [11, 45]. Both opinion leaders and the policy environment are recognized as influential dissemination determinants and represent important targets for strategies aimed at facilitating the spread of initiative information [37]. For example, schools that already have a physical activity policy are likely to have prior exposure to the existing knowledge, which can facilitate understanding and receptivity to the information being disseminated. However, schools with existing physical activity policies are often lacking in practical guidance on how to implement them [46]. Positioning *TransformUs Secondary* as a vehicle to operationalize these policies provides a legitimate channel for dissemination and uptake.

Overall, this study demonstrated that national-level dissemination is complex and challenging, underscoring the need to recognize both partnership and dissemination strategies as a central component of scale-up [5, 37, 47]. Research–practice partnerships are central to dissemination by providing access to established infrastructure and networks, yet they simultaneously introduce structural constraints (e.g. competing priorities and limited capacity) [48]. Thus, over-reliance on partners without adequate support may undermine engagement, underscoring the need for scale-up planning that balances responsibilities among researchers, partners, and system-level stakeholders during this initial stage of scale-up [40]. To date, guidance provided by the current literature on dissemination remains considerably less developed compared with those available for implementation [5, 37, 49]. Findings from this study and our prior work [13] indicate that existing dissemination strategy frameworks which focus primarily on developing and distributing materials [7], may not fully capture the range of strategies identified, highlighting the need to broaden theoretical guidance. Moreover, dissemination factors in this study were driven by target audience, channels, and intermediary organization domains, which are not included in existing determinant frameworks (e.g. the Ecological Framework [16]), highlighting the need for dissemination-specific determinants frameworks. Collectively, these findings support calls for flexible frameworks that help researchers and stakeholders adapt dissemination strategies during scale-up, as traditional step-by-step frameworks cannot capture real-world uncertainty and adaptation [40].

Strengths of this study include mapping dissemination strategies and pathways using existing frameworks, which enhances shared terminology and understanding of dissemination processes across partners, while also highlighting gaps in existing frameworks and the literature. The study provides novel qualitative insights into the types of strategies that can be designed, the contextual factors influencing their use, and the mechanisms through which key strategies were perceived to affect outcomes. These findings are important for informing researchers in how to develop their dissemination strategies beyond reliance on intuition or prior precedent [39]. Limitations include the non-participation of six organizations, which may have limited the breadth of perspectives captured. Participants’ existing relationship with *TransformUs* may have influenced the openness of their responses; however, anonymity and confidentiality were assured, and interviews were conducted by the lead author, a PhD candidate independent of the core *TransformUs* research team, to encourage candid discussion.

## Conclusion

The findings provide novel insights into how national dissemination of a school-based physical activity initiative operated across diverse partners. Dissemination strategies that navigated real-world complexity were identified. Dissemination was complex and context-dependent, involving active, targeted, and locally tailored engagement beyond merely sharing initiative materials. Future studies in the design and testing of dissemination strategies are needed. In practice, researchers and partners should strengthen interactive, capacity-building engagement, establish clear dissemination pathways, and sustain ongoing promotion while addressing barriers that hinder partners’ dissemination efforts. Policies should support frameworks that help organizations navigate structural challenges and provide effective channels for disseminating initiatives to schools.

## Supplementary Information

Supplementary Material 1.

Supplementary Material 2.

Supplementary Material 3.

Supplementary Material 4.

Supplementary Material 5.

## Data Availability

The datasets generated and analysed during this study are not publicly available due to ethical and confidentiality considerations. Data are stored in encrypted form and can only be accessed by researchers approved under the relevant Deakin Human Research Ethics Committee protocols (Project ID: 2021-269; HEAG-H 32_2021), approved on 20 September 2021. Requests for access to the data should be directed to the corresponding author at jo.salmon@deakin.edu.au.
