# Supplementary material for: Dissemination strategies for the national scale-up of a school-based physical activity initiative: insights from education and health sector partner organizations
Source: BMC Public Health. 2026 Jul 14;26:2880. doi: 10.1186/s12889-026-28517-4 (PMC13647789; doi:10.1186/s12889-026-28517-4)
Supplement: Supplementary file 3 [file 12889_2026_28517_MOESM3_ESM.docx]

Supplementary file 3. Interview schedule

Instructions – to be read to the participant at the start of the interview.

Thank you for coming, I am a PhD candidate involved in the *TransformUs Secondary* Project.

You have been invited to participate in this interview as you represent an organisation that helped dissemination and scale up of *TransformUs Secondary* in your state. As a reminder, *TransformUs Secondary* is a whole-of-school initiative that aims at increasing physical activity and reduce sedentary behaviour among adolescents. *TransformUs* initiative provides professional learning and free resources for teachers to aid them in delivering the initiative in the classroom, such as active breaks, active lessons, and active homework.

Our discussion should last no more than 30 minutes. Questions will relate to your experiences and thoughts of disseminating and scaling up the *TransformUs Secondary*, including any barriers or enablers you might have faced. There are no right or wrong answers, so please speak freely and honestly about your current experience.

This interview will be audio-recorded, transcribed, and coded anonymously for analysis. Your name will not be used in any of the published material, and your responses will remain unidentifiable.

I want to start by asking you a few basic questions about yourself.

| Question | Note/Prompt/follow-up questions |
| --- | --- |
| **Descriptive**   1. What is your current job role? 2. How long have you held this position? 3. What influenced your organisation's decision to help with the dissemination and scale-up of *TransformUs Secondary*? | Follow up  How does it relate to the dissemination and scale-up of *TransformUs Secondary*?  Follow up  What previous experience, if any, have you had in similar dissemination or scale-up efforts?  Follow up  Was it based on perceived initiative benefits, alignment with organisational goals, or external recommendations? |
| **Dissemination**  Now, we move the dissemination section, dissemination is defined as spreading information or materials about *TransformUs Secondary* to schools and teachers using various channels (such as website, newsletters, teacher networks) to increase their awareness, attitude, and motivation to adopt the initiative. | |
| 5. Have you been involved in disseminating *TransformUs Secondary?*   *Barriers/Enablers*   6. What challenges did you face while disseminating the initiative? and what helped you disseminate the initiative?   *Dissemination strategies*   7. How did your organisation encourage schools and teachers to adopt *TransformUs*? 8. Could you share examples of what strategies worked well and why? 9. Did you attend the *TransformUs Secondary* webinar launch in your state/territory? 10. Did you receive and/or use the Partner toolkit? | Follow up  Can you describe your role in disseminating *TransformUs Secondary*?  Follow up   - Who did these strategies? - How were these strategies implemented? (action, frequency)   Follow up   - Did you find it helpful? What aspect most useful, why? - If not, why not?   Follow up   - If yes, how did you use it, and what impact did it have? - If not, was there a reason, and how could it have been more useful? |
| **Scale-up**  Now, we move to the scale-up section. Scale-up is defined as expanding the reach and sustainability of the initiative by facilitating its integration and implementation into schools, through mechanisms such as policy, securing funding, PL, etc.  Unlike dissemination, which focuses on spreading awareness and information about *TransformUs*, scale-up involves structural and long-term strategies (maintenance) to ensure the initiative becomes an embedded and sustainable part of school systems. | |
| 11. Has your organisation played a role in supporting the scale-up (Examples: grants, professional development, other fundings) of *TransformUs Secondary*? If yes, what are the strategies?   *Barriers/Enablers*   11. What were the challenges? What condition facilitated these actions? | Follow up   - Who did these strategies? - How were these strategies implemented? (action, frequency) |
| **Recommendations**   13. Based on your experiences over the last year, can you recommend ways to:  - Increase uptake of the initiative among teachers and/or schools - Improve implementation of the initiative in practice - likelihood it becomes embedded as part of the schools' routine practice.  14. Do you have any further comments you would like to make that we haven't covered? | Note  Select as appropriate based on previous answers. |

Thank you to the participant.
