# Supplementary material for: Dissemination strategies for the national scale-up of a school-based physical activity initiative: insights from education and health sector partner organizations
Source: BMC Public Health. 2026 Jul 14;26:2880. doi: 10.1186/s12889-026-28517-4 (PMC13647789; doi:10.1186/s12889-026-28517-4)
Supplement: Supplementary file 4 [file 12889_2026_28517_MOESM4_ESM.docx]

Supplementary file 4. Themes and sub-themes

| No | Theme | Sub-theme | Sub-theme description |
| --- | --- | --- | --- |
| 1. | Dissemination strategies of *TransformUs Secondary* | Types | Dissemination strategies encompassed message and material development, education material distribution, educational meetings, and network and liaison utilization for dissemination |
|  |  |  |  |
|  |  | Pathways | Different sectors targeted distinct audiences: the education sector focuses on school staff, while the health sector engages intermediaries who liaise with schools. Both used tailored messages and channels |
|  |  |  |  |
|  |  | Frequency | Dissemination frequency varied: sporadic emails, regular newsletters, frequent professional development, and permanent placement on websites and secondary school physical education textbook |
|  |  |  |  |
| 2. | Dissemination and scale-up barriers and enablers | Factors to teacher engagement and adoption | Perceived initiative complexity, low relevance, teachers’ reluctance to take on extra tasks, and time constraints limit engagement and adoption, while perceived benefit support adoption. |
|  |  |  |  |
|  |  |  |  |
|  |  | Factors to partner dissemination | Dissemination is limited by organizational challenges such as bureaucracy, role boundaries, and capacity, while organisational alignment, existing network, and dissemination toolkits facilitate partner dissemination. |
|  |  |  |  |
|  |  | Factors to scale-up | Lack of funding and competing priorities influence initiative dissemination |
|  |  |  |  |
| 3. | Mechanisms for effective dissemination  & scale-up strategies | Reflective motivation | Strategic framing and simple packaging are crucial to improve perception toward the initiative and motivate teacher to engage and adopt the initiative. |
|  |  |  |  |
|  |  | Social opportunity | Leveraging school leaders and school policies are needed to legitimate and advocate the initiative that encourage engagement and adoption among school community |
|  |  |  |  |
|  |  | Physical  opportunity | Digital-based dissemination is vital to enabling teachers access to resources to expand reach |
|  |  |  |  |
|  |  | Physical capability | Education-based dissemination is important to build teacher skill that support adoption |
