# Supplementary material for: Dissemination strategies for the national scale-up of a school-based physical activity initiative: insights from education and health sector partner organizations
Source: BMC Public Health. 2026 Jul 14;26:2880. doi: 10.1186/s12889-026-28517-4 (PMC13647789; doi:10.1186/s12889-026-28517-4)
Supplement: Supplementary file 5 [file 12889_2026_28517_MOESM5_ESM.docx]

Supplementary file 5. Supporting quotes for dissemination strategy types

| Dissemination strategies | Dissemination activities | Illustrative Quote | Participant Role & Organization |
| --- | --- | --- | --- |
| Develop promotional messages and educational materials | Developing promotional flyers | *“We're developing our own resources. A professional teaching organization are working with us and I have asked them to create resources for secondary schools to get kids active. There will be some really simple one page exercises, like what to do at lunchtime and how to line markings for courts. And one of those one-page flyers will say, and here are some excellent resources.”* | Participant 1, organization A |
|  | Promotion developed by media team | *“We do have a media team that look at that as their bread and butter.”* | Participant 5, organization B |
|  | Help curriculum alignment | *“I do a lot of work in supporting the development of resources from an advisory perspective, so I give a curriculum perspective to that. For instance, if TransformUs Team ever reached out, we obviously have reasonable regular contact and said Chris, can you look at this just from a curriculum perspective, whatever it might be? Absolutely I do that.”* | Participant 9, organization A |
| Distribute messages and materials | Distribute newsletters | *“For example, when TranformUs Team were looking for volunteers, schools in secondary to trial the initiative, though, after 20 volunteer schools, I actually put that out in the newsletter.”* | Participant 2, organization C |
|  |  | *“So for TransformUs, yes… We have our newsletter that reaches thousands of people.”* | Participant 10, organization B |
|  |  | *“We've got newsletters that are targeted to both of those sorts of audiences, so to community as well as stakeholders”* | Participant 5, organization B |
|  | Upload the initiative on existing teacher digital platform | *“We got, like, an e-Learn. It's like a website where we put our resources up, so that all teachers in the state can access. So, it's kind of ongoing promotion. I put it (TransformUs Secondary) on our e-learn website.”* | Participant 3, organization A |
|  |  | *“For example, this is our body brain being materials. So this is some of the physical literacy materials that we provide for teachers. So, it (the TransformUs Secondary initiative) is under the Physical literacy tab”* | Participant 6, organization A |
|  | Email blasts to teachers, colleagues | *“I would have just copied and pasted into an email, so, that would have gone out to professional teaching organization members. It would have also gone out to my colleagues and teachers contact list.”* | Participant 3, organization A |
|  | Secondary school PE textbook | *“I've actually put a TransformUs article in a year 9 and 10 health book that's going to be released very shortly for the new physical education and health curriculum. So, I'm trying to promote TransformUs in a textbook.”* | Participant 2, organization C |
| Conduct teacher and stakeholder educational meeting | Teacher professional learning, webinar, conference | *“We have this one (professional development) initiative that we go and train whole schools. There's some activity from TransformUs that we do. So, when we're doing that, I always promote TransformUs, and we always have it up on the board as one of the resources that we've used.”* | Participant 3, organization A |
|  | Showcase to stakeholders, stakeholders meetings | *“Now we run networks across the catchment, which we can have a lot of people attending. So, we've disseminated that information that we received about TransformUs Secondary.”* | Participant 10, organization B |
| Leverage network and liason for dissemination | Leverage network | *‘So, we do a lot of partnering with them on various sort of initiatives and efforts. And they're often quite supportive of sharing messaging through their channels as long as it's consistent with what their remit is as well.’* | Participant 5, organization B |
|  | Liaison for dissemination | *‘We don't directly have schools attend network catchment. I should say it's the partners like local government as who then disseminated to the schools or the community health who work with the schools.’* | Participant 10, organization B |
