# Supplementary material for: Dissemination strategies for the national scale-up of a school-based physical activity initiative: insights from education and health sector partner organizations
Source: BMC Public Health. 2026 Jul 14;26:2880. doi: 10.1186/s12889-026-28517-4 (PMC13647789; doi:10.1186/s12889-026-28517-4)
Supplement: Supplementary file 6 [file 12889_2026_28517_MOESM1_ESM.docx]

Supplementary file 1. Dissemination toolkit for partners (example pages)
